# Enhanced inulin production by hairy root cultures of *Cichorium intybus* in response to Pi and Fe starvation

**DOI:** 10.22099/mbrc.2021.38031.1527

**Published:** 2021-06

**Authors:** Somayeh Tabatabaee, Forough Sanjarian, Tahmineh Lohrasebi, Mahsan Karimi

**Affiliations:** 1 Department of Plant Bioproducts, National Institute for Genetic Engineering and Biotechnology, Tehran, Iran; 2 Entomology, Plant Pathology and Nematology Department, University of Idaho, Moscow, ID 83844-2329, USA

**Keywords:** Cichorium intybus, Inulin, Agrobacterium rhizogenes, Phosphate starvation, Iron starvation

## Abstract

*Cichorium intybus* is rich in inulin and has several pharmacological applications. Hairy roots culture is a valuable biotechnological tool used to produce plant secondary metabolites. *Agrobacterium rhizogenes*-mediated genetic transformation of chicory to hairy roots was investigated using Agrobacterium Strains A4, A13, A7, and ATCC15834. Several hairy roots were tested, from which 17 lines were selected based on their fast-growing characteristics. Results of PCR analysis revealed foreign DNA integration into the selected transgenic hairy root lines. Finally, four Adventitious roots that contained the highest ratio of total sugar to total weight (µg/gr DW), were selected. This study investigated the effects of various levels of minerals and sucrose on the production of inulin in Cichorium hairy root culture. Different levels of sucrose, phosphate (Pi) and Iron (Fe) were evaluated, separately. It was found that an increase in sucrose levels from 3 to 5% could decrease the root growth; however, 60 g/l sucrose remarkably enhanced the inulin production rate in all the examined lines. The highest biomass was achieved by applying 3.75 mM Pi but it ended in the decreasing the inulin content per unit weight. In contrast, the highest inulin accumulation and the lowest amount of biomass were observed in 0.5 mM Pi. Fe starvation caused the biomass decrease and a significant increase in inulin accumulation. Results of this study suggest a successfully optimized culture medium to initiate the induction of *Cichorium intybus* hairy root cells to produce inulin as a valuable medicinal secondary metabolite.

## INTRODUCTION

*Cichorium intybus*, known as chicory, is a diploid plant species that belongs to the *Asteraceae* family which is mainly found in Europe and Asia [1]. Different parts of this plant are famous to have anthelmintic, antimalarial, hepatoprotective, antidiabetic, anti-inflammatory, and tumor-inhibitory effects [2, 3]. Nowadays, most of the chicory studies, focus on the properties of chicory roots as a main source of inulin which is a plant storage polysaccharide that belongs to a group of non-digestible carbohydrates called fructans. Inulin rose to fame for its extraordinary applications in food and pharmaceutical industries [4, 5]. 

Biotechnological methods such as Transgenic hairy root culture and *in vitro* regeneration have revolutionized the metabolic engineering and the production of the secondary metabolites in medicinal plants [6]. Induction of hairy roots in herbal plants by soil borne bacterium like *Agrobacterium rhizogenes* provides an appropriate system for synthesis of valuable secondary metabolites such as pharmaceutical compounds. Induced hairy roots caused rapid growth, and elevated genetic and biochemical stabilities. Italso, gives the ability to grow in hormone-free media, and the potential to synthesize a variety of chemical compounds [7, 8].

Sucrose is an important carbon and energy source for most Adventitious root cultures, and it is proved that its concentration has obvious influence on growth levels and, also the yield of secondary metabolites. An increase of sucrose concentration in the medium to above the normal range of 2–3%, could stimulate the production of phenols and flavonoids in cultures of *Echinacea angustifolia* [9].

Furthermore, Mineral concentrations in the medium can also affect the enhancement of hairy root induction, the regulation pathways through *Agrobacterium*, and the accumulation of important bioactive compounds [10-12]. There are several reports reporting that changing the Pi or Fe concentrations could enhanced secondary metabolites [13, 14]. It was also shown that decreasing the Pi concentration favored the biomass production in the Adventitious root culture of *P. heterophylla* [15]. Moreover, adding Fe_2_O_3_ nanoparticles, enhanced the production of hyoscyamine and scopolamine in the hairy root culture of *Hyoscyamus reticulatus* L [16]. The aim of the present study was to induce the hairy root cultures of *Cichorium* *intybus* with Pi and Fe starvation and assaying its effects on inulin production.

## MATERIALS AND METHODS

The seeds of *Cichorium intybus* (Pakan Bazr Isfahan, Isfahan, Iran) were sterilized by submerging in 70 % (v/v) ethanol for 2 min and then, 70 % (v/v) sodium hypochlorite solution for 15 min. Next, they were cultured on MS basal medium under a 16/8 h (day/night) photoperiod at 25°C in a growth chamber [17]. For hairy root induction, *Agrobacterium rhizogenes* strains A4, ATCC15834, A7, and A13 were applied for the infection studies. 3-week-old leaves, cotyledons, and roots from 7-day old seedlings were used as explants. Wounded explants were pre-cultured in MS liquid medium containing 30 mg/L sucrose with and without 100 μM acetosyringone for 30 min, then plunged in bacterial suspension (OD_600_ = 0.6) for 2 and 10 min, respectively. The explants were briefly dried and then incubated in a dark growth chamber at 25±2°C on agar-solid MS medium for 3 days. For the elimination of *A.* *rhizogenes*; the explants were transferred to MS medium with the aid of 200 mg/L cefotaxime. 

13 to 15 days after the transformation, putative hairy roots were appeared in the injured parts of the explants. Finally, 85 lines of the induced hairy roots were observed. Explants were cut off from the tissues and sub-cultured every 20 days in a hormone-free MS medium with the aid of cefotaxime at 25 °C and in dark condition. The growth rate and the filling of the plate up to 50% of the whole content, were monitored in a two-week timeframe. Among 85 tested lines, 17 lines with the highest growth, were selected for the remainder of the study. Genomic DNA was extracted from the selected transformed hairy root lines using a modified CTAB protocol method [18]. PCR amplification of *rol*B gene was performed using 5′-GCT CTT GCA GTG CTA GAT TT-3^'^ as forward primer and 5′-GAA GGT GCA AGC TAC CTC TC-3' as reverse primer, respectively. 4 lines with the highest total inulin were selected for the final study. In order to select the best lines, 2 g of hairy roots were added to 50 ml liquid MS medium and 250 μl cefotaxime (pH 5.8). They were stirring in an orbital shaker (90 rpm), at 25° C in the dark. Hairy roots were weighed every 10 days and up to 30 days after cultivation date to find out the biomass (g/L, FW) and total sugar (g/L). 

Fresh chicory hairy roots were used to extract water-soluble carbohydrates. The hairy roots were chopped and diluted with sterilized distilled water (1:5). The mixture was then blended with hydrochloric acid 0.1 N to hit the pH 5. The final mixture was heated at 60± 2°C for 1 h, under continuous stirring (5 rpm). After this period, solid and liquid phases were separated by filtration (0.45 μm). The concentrated extracts were then centrifuged at 4500 g for 10 min and 1 ml of the supernatant was diluted to 100 ml.

Total carbohydrates in the  water-soluble components, extracted from chicory hairy roots, were determined by phenol sulfuric acid method [19]. Concentrations of reducing sugar of the hairy roots were determined by dinitrosalicylic acid method (DNS). The amount of reducing sugars were measured by spectrophotometer (SPECORD 50/PLUS) at 530 nm and then, analyzed by using glucose standard curve. Degree of inulin polymerization was obtained by dividing the total sugar weight by the total weight of the reducing sugars.

Four adventitious roots that contained the highest total sugars were selected and inoculated into MS medium supplemented with 3, 4, 5, and 6% (w/v) sucrose, respectively. Then, 0.2 g hairy roots were cultured in 50 mL of the liquid medium and incubated at 25°C (pH 5.8) in an orbital shaker (90 rpm), in the dark. The growth rate (FW) and the total carbohydrates weights were measured after 2 weeks.

After optimizing all the mentioned parameters;1 line was selected based on the highest rate of total sugar to the total weight. The effect of Pi source on inulin production rate, was investigated in hairy roots that were grown in MS medium, supplemented with different levels of Pi and Fe. The hairy roots were grown in MS for 10 days and then were transferred to MS medium that didn’t contain neither Fe nor Pi. In order to investigate the effect of Pi, 0.5, 0.83, 2.5, 3.75 mM KH_2_PO_4_ was added, respectively and were compared to control medium (which had 1.25 mM KH_2_PO_4_). To investigate the effect of Fe on inulin production, MS medium with and without FeSo_4_ were compared. 

A completely randomized design was used for all the treatments with three replications. Data were statistically analyzed using the SAS software. The mean separations were carried out using Duncan’s multiple range tests and the significance was determined at the level of 5%.

## RESULTS

In the present study, the efficacy of bacterial strains, different explants, and different exposure times (2 and 10 min, respectively) were investigated in hairy root induction. After two weeks of co-cultivation, hairy roots appeared only in the hypocotyl explants. Also, the difference in the induction time was not significant. The amplification of *rol*B genes by PCR confirmed the transgenic nature of the roots (Fig. S1). The highest transformation frequency was 60% that was achieved using A7 strain, followed by 50% which was reported for the treatment that used ATCC15834, and then 40% transformation frequency that was gained by using A13. Statistical analysis showed no significant difference between these strains. A4 strain did not show any induced hairy roots. 

**Figure 1:**
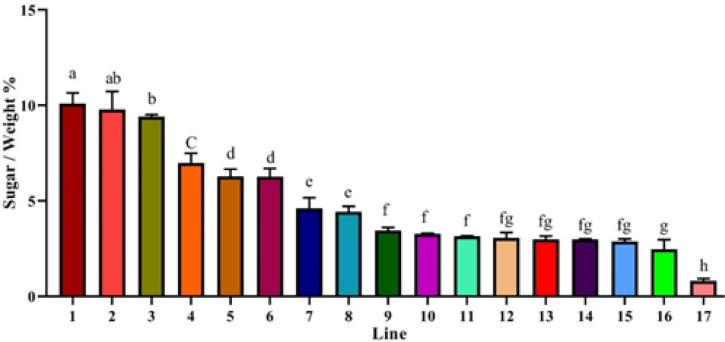
The carbohydrate content of the hairy root tissues in the 1, 2, 3, 4 lines was significantly higher than the other lines. The different letters denote a statistically significant difference at P≤0.05, as determined by Duncan’s multiple range tests

Finally, 17 lines were selected based on their growth rates and 50% filling of the plate within a two-week timeframe. These lines were then transferred to liquid medium for further analysis. After two weeks, the ratio of total carbohydrate to total weight was obtained and four lines with the highest ratios were selected for the subsequent experiments (Fig. 1 and Fig. S2).

**Figure 2:**
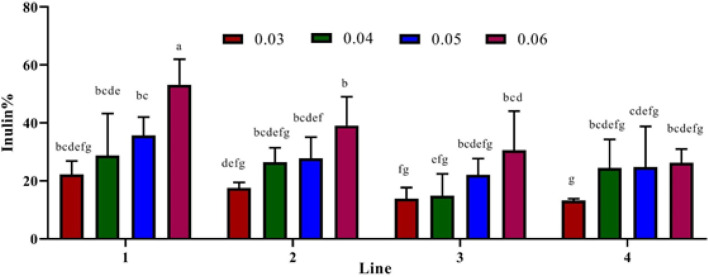
Effects of sucrose at various concentrations on inulin content. The different letters denote a statistically significant difference at P≤0.05, as determined by Duncan’s multiple range tests

The effect of adding different levels of sucrose (3-6%) in hormone-free MS medium was evaluated based on the ratio of dry weight to fresh weight (DW/FW) of hairy roots. In all the lines, hormone-free MS medium containing 3% sucrose favored the DW/FW ratios; the highest DW/FW (5.7%) of hairy roots was obtained in line 1 (Fig. S3). In this study, maximum inulin content (53%) was seen in Line 1 where the medium was supplemented with 6% sucrose. According to Figure 2, inulin accumulation increased by the elevation of sucrose levels, while minimum inulin accumulation was observed in line 4 with 3% sucrose.

**Figure 3:**
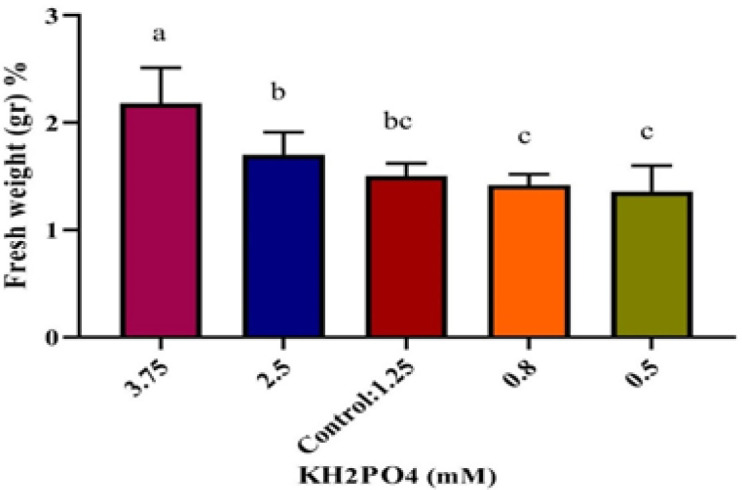
Effects of phosphate concentrations on hairy root fresh weight on Line 1. The different letters denote a statistically significant difference at P≤0.05, as determined by Duncan’s multiple range tests

**Figure 4:**
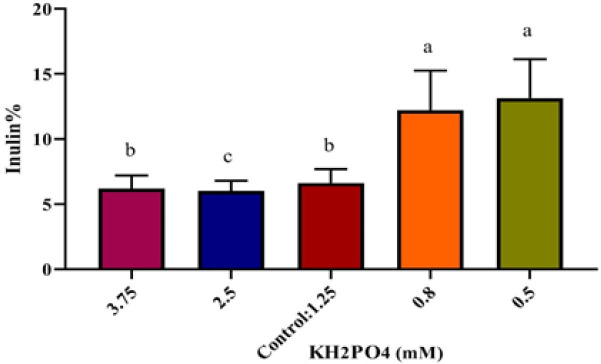
Effects of phosphate concentrations on inulin accumulation on Line 1. The different letters denote a statistically significant difference at P≤0.05, as determined by Duncan’s multiple range tests

In this study, assaying Pi concentrations effectiveness was performed in four treatments of hairy roots: two of them contained more than 1.25 mM Pi (2.5, and 3.75 mM, respectively) and two of them contained less than 1.25 mM Pi (0.83, and 0.5 mM, respectively) while MS medium had 1.25 mM Pi concentration. Results of these tests showed that in 2.5 mM Pi, fresh weight of hairy roots did not change significantly compared to the control. However, in 3.75 mM Pi, fresh weight increased significantly compared to control and reached 2.17 g. Although, fresh weight decreased in hairy roots that were grown in Pi deficient medium (0.5- and 0.8-mM Pi, respectively), but this decrease was not significant. In contrast, the highest inulin percentages were achieved in 0.5- and 0.8-mM phosphate, respectively but no significance difference was found in inulin percentages between 3.75 mM Pi and the control. The highest and the lowest amounts of inulin were 13.15% and 6.08%, respectively which was related to 0.5 mM Pi and 2.5 mM Pi, respectively (Fig. 3 and 4).

Results of inulin accumulation assays indicated that Fe starvation had a significant increase in *C. intybus *hairy roots in comparison with hairy root growth in the MS containing 9.9% and 6.6% inulin, respectively. However, biomass analysis demonstrated a significant biomass decrease in Fe starvation medium (1.0g/100 ml) compared to the MS (1.53 g/100 ml) (Table 1).

**Table 1:** Effects of iron starvation on hairy root fresh weight and inulin accumulation on Line 1

	**Control**	**Fe Starvation**
**Fresh Weight(g)**	1.53±0.49^a^	1±0.41^b^
**Inulin%**	6.6±0.86^b^	9.9±0.091^a^

The different letters denote a statistically significant difference at P≤0.05, as determined by Duncan’s multiple range tests.

## DISCUSSION

According to the results achieved in this study, the highest hairy roots induced by hypocotyl explants, in the hormone-free medium without acetocyringone, were A7 (60%), ATCC15834 (50%), and A13 (40%) *A*. *rhizogenes *strains, respectively. Accordingly, in Kabirnataj et al (2016) study *A. rhizogenes* strains A13, showed high-efficient transgenic hairy root induction in chicory [20]. Furthermore, genetic transformation of two species of chicory that employed *A. rhizogenes* K599 harboring p35SGFPGUS+ plasmid was evaluated and the transformation rate of about (23.1%) was reported in leaf explants of *C. intybus* [21].

The rate of biomass growth was found to be directly correlated with sugar utilization rat Sucrose, can synthesize cell constituents , provide energy for cell growth ,and increase the accumulation of secondary metabolites and polysaccharides [11]. In our study, the largest amount of the total sugar was recognized in culture supplemented with 6% sucrose. It was reported that, in growth of Cichorium hairy roots, 3% sucrose was optimum while, inulin accumulation increased by the elevation of sucrose levels. Similarly, Yin et al (2013) investigated the induction ability of Adventitious root on *Pseudostellaria heterophylly* and also the effects of sucrose concentration on biomass increase and metabolites accumulation. They found that in the medium with 4 % sucrose, optimum biomass was achieved, and also the content of saponin and polysaccharides were highest in this condition [15]. 

Pi is one of the main vital macronutrients for plant growth and development [22]. Our results illustrated an accumulation of inulin upon Pi starvation. This is in agreement with the report of Liu et al (2018) that an increase in the Pi concentration in the culture medium improved the growth parameters but also decreased the accumulation of specific secondary metabolites, such as phenolic acids and tanshinones on hairy roots of *Salvia miltiorrhiza* and *Salvia* *castanea* [23].

Recent studies have revealed that any changes occurring in roots during Pi starvation are an outcome of complex interactions between Pi and other nutrients, especially Fe [24]. The results of our study indicated that in Fe starvation treatment, inulin accumulation significantly increased, but the biomass decreased remarkably which can be due to the role of Fe in respiratory systems. During the Fe- starvation, cytochrome pathway was restricted and hence the decrease in biomass could be expected [25]. According to the investigation on the responses of sugar beetroots to Fe deficiency, it was indicated that iron deficiency causes an approximately 50-fold enhancement in carbon assimilation in the cytosol through the increase in phosphoenol pyruvate carboxylase activity [26]. 

Results of the current study demonstrated an efficient *A. rhizogenes*-mediated transformation for the foundation of hairy root cultures. The accumulation of the total inulin increased considerably in response to high sucrose levels. This study will help to elucidate the specific roles of mineral concentrations in the medium of *Cichorium intybus* for inulin production in hairy roots. Further studies using bioreactors for a scaled-up inulin accumulation at the commercial level will be required.

## Supplementary Materials


