# Supplementary material for: Enhanced inulin production by hairy root cultures of Cichorium intybus in response to Pi and Fe starvation
Source: Mol Biol Res Commun. 2021 Jun;10(2):85–91. doi: 10.22099/mbrc.2021.38031.1527 (PMC8310654; doi:10.22099/mbrc.2021.38031.1527)
Supplement: Supplementary file 1 [file mbrc-10-85-s001.pdf]

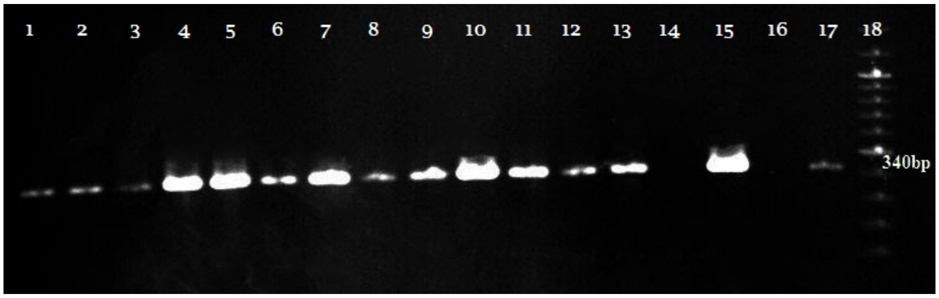

Figure S1: PCR amplification of the *rol*B gene in hairy roots of *Cichorium intybus*. 1-13: Hairy roots, 14: Normal root, 15: *A. rhizogenes* plasmid DNA (positive control), 16: negative control, 17: hairy root lines, 18: DNA Ladder (100 bp, Yektataghiz, Iran).

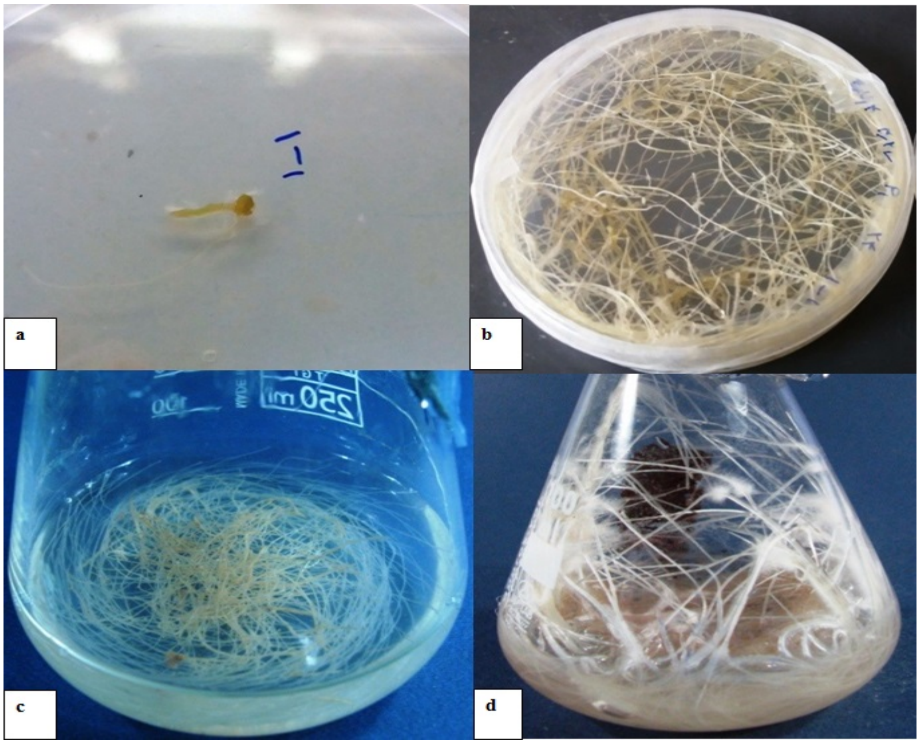

Figure S2: Hairy roots propagation. **a,b** Single-line which grown and filled up 50% of the solid medium plates in two weeks after selection; **c,d** Selected hairy root in liquid MS medium. (a-d:1x).

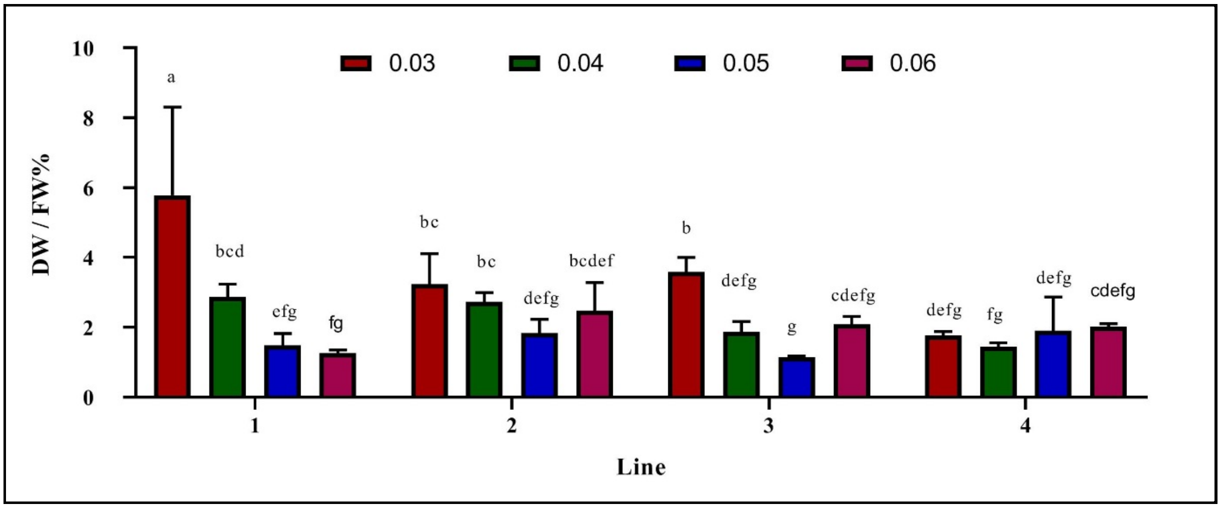

Figure S3: Effects of sucrose concentrations on dry weight /fresh weight percent in select lines. The different letters denote a statistically significant difference at P≤0.05, as determined by Duncan's multiple range tests.
